# Supplementary material for: Chalcone-Synthase-Encoding RdCHS1 Is Involved in Flavonoid Biosynthesis in Rhododendron delavayi
Source: Molecules. 2024 Apr 17;29(8):1822. doi: 10.3390/molecules29081822 (PMC11054853; doi:10.3390/molecules29081822)
Supplement: Supplementary file 1 [file molecules-29-01822-s001.zip › Table S1.pdf]

Supplementary Table S1 List of primers used in this study

**Primer name**	**Primer sequence (5'→3')**
RdCHS1-gene cloning-F	AAATGGCTCCTGGTGGTGTGTC
RdCHS1-gene cloning- R	GCTGCCATTTCCAGTATCAAC
RdCHS1-qRT-F	TCAGTGGAGGAATTCCGAAAG
RdCHS1- qRT-R	TTAACGCAGTTAGCAGGAGTAG
*Rd CHS1*F-32	CGGGATCCATGGCTCCTGGTGGTGTG
*Rd CHS1*R-32	CCCAAGCTTTCAGTGAGCCGGAACGCT
*Rd CHS1*F-121	GCTCTAGAATGGCTCCTGGTGGTGTG
*Rd CHS1*R-121	CGGGATCCTCAGTGAGCCGGAACGCT
NtCHS-qRT-F	TGACACCCACTTGGATAGTTTAG
NtCHS-qRT-R	CGACCTCTGGAATTGGATCAG
NtCHI-qRT-F	CTTTTCTCGCCGCTAAATG
NtCHI-qRT-R	TTTCTGCCACCTTCTCTG
NtF3'H-qRT-F	AGGCTCAACACTTCTCGT
NtF3'H-qRT-R	CATCAACTTTGGGCTTCT
NtF3'5'H-qRT-F	CGCACTACCATACTTAGGAGCCAT
NtF3'5'H-qRT-R	CAGCATCAGGAGTAGAAGCAACAG
NtDFR-qRT-F	AACCAACAGTCAGGGGAATG
NtDFR-qRT-R	TTGGACATCGACAGTTCCAG
NtANS-qRT-F	TGGCGTTGAAGCTCATACTG
NtANS-qRT-R	GGAATTAGGCACACACTTTGC
NtUFGT-qRT-F	GAGTGCATTGGATGCCTTTT
NtUFGT-qRT-R	CCAGCTCCATTAGGTCCTTG
NtTubA1-qRT-F	CTCCTATGCTCCTGTCATTTC
NtTubA1-qRT-R	GGCGAGGATCACACTTAAC
NtAN2-qRT-F	GAAGAAAGGTGCATGGACTG
NtAN2-qRT-R	TCTGCAGCTCTTTCTGCATC
NtAN1a- qRT-F	ACCATTCTCGAACACCGAAG
NtAN1a- qRT-R	TGCTAGGGCACAATGTGAAG
NtAN1b- qRT-F	CTTGAACACTTCTCAAACCGA
NtAN1b- qRT-R	TGCTAGGGCACAATGTGAAG
β-actin -qRT-F	GGCCGTGGTAGTGAACATATAA
β-actin -qRT-R	GTATGCCCTTCCTCATGCTATC
NtTubA1-qRT-F	CTCCTATGCTCCTGTCATTTC
NtTubA1-qRT-R	GGCGAGGATCACACTTAAC
Rdactin1-qRT-F	GTATGCCCTTCCTCATGCTATC
Rdactin1-qRT-R	GGCCGTGGTAGTGAACATATAA
RdCHS2-gene cloning-F	AAAATCCCAGTATGTGCGAGTA
RdCHS2-gene cloning- R	CGTATGAGCGTCCACGAAGT
RdCHS3-gene cloning-F	ATGGTCAGGATAGAGGATGTTC
RdCHS3-gene cloning- R	TCCACATGAGCGTCCACGAA

* Restriction enzyme site are underlined
