# Supplementary material for: Chalcone-Synthase-Encoding RdCHS1 Is Involved in Flavonoid Biosynthesis in Rhododendron delavayi
Source: Molecules. 2024 Apr 17;29(8):1822. doi: 10.3390/molecules29081822 (PMC11054853; doi:10.3390/molecules29081822)
Supplement: Supplementary file 1 [file molecules-29-01822-s001.zip › Table S2.pdf]

Table S2 The anthocyanin profiles in acidic MeOH-H_2O extracts of the *RdCHS1* transgenic *Arabidopsis*

Seedling	Cyanidin	Pelargonidin
Wild Type	177.06[a]	95.19[a]
Mutant	N/D	N/D
RdCHS1-1	121.69[b]	154.55[b]
RdCHS1-3	138.02[b]	144.93[b]
