# Supplementary material for: Chalcone-Synthase-Encoding RdCHS1 Is Involved in Flavonoid Biosynthesis in Rhododendron delavayi
Source: Molecules. 2024 Apr 17;29(8):1822. doi: 10.3390/molecules29081822 (PMC11054853; doi:10.3390/molecules29081822)
Supplement: Supplementary file 1 [file molecules-29-01822-s001.zip › Table S3.pdf]

Table S3 HPLC-DAD and HPLC-ESI-MS analysis of anthocyanin in acidic MeOH-H_2O extracts of the wild-type *Arabidopsis* and *RdCHS1* over-expressing lines

Peak number	Identifacation/tentative identification	Retention time (min)	λmax (nm)	ESI-MS (m/z)	References
1	Cyanidin 3-*O*-[2″-*O*-(xylosyl) 6″-*O*-(*p*-*O*-(glucosyl) *p*-coumaroyl) glucoside] 5-*O*-[6″″-*O*-(malonyl) glucoside]	45.565	266<br>526	287.1[Cy+H]$^+$<br>1137.4[M+H]$^+$	Takayuki Tohge, Yasutaka Nishiyama et al., 2005 [1]
2	Cyanidin 3-*O*-[2″-*O*-(6‴-*O*-(sinapoyl) xylosyl) 6″-*O*-(p-*O*-(glucosyl)-*p*-coumaroyl) glucoside] 5-*O*-(6″″-*O*-malonyl) glucoside	47.720	294<br>534	287.0[Cy+H]$^+$<br>1343.2[M+H]$^+$	Stephen J. Bloora, Sharon Abrahamsb., 2002 [2]
3	Pelargonidin derivatives	56.831	282<br>527	271.2[Pg+H]$^+$	
4	Pelargonidin derivatives	57.605	284<br>534	271.3[Pg+H]$^+$	

Reference list for Tables S3

54. Tohge T, Nishiyama Y, Hirai MY, Yano M, Nakajima J, et al. (2005) Functional genomics by integrated analysis of metabolome and transcriptome of Arabidopsis plants over-expressing an MYB transcription factor. Plant J 42: 218-235.

55. Stephen J. Bloora, Sharon Abrahamsb (2002) The structure of the major anthocyanin in Arabidopsis thaliana. Phytochemistry 59: 343-346.
