# Supplementary material for: Chalcone-Synthase-Encoding RdCHS1 Is Involved in Flavonoid Biosynthesis in Rhododendron delavayi
Source: Molecules. 2024 Apr 17;29(8):1822. doi: 10.3390/molecules29081822 (PMC11054853; doi:10.3390/molecules29081822)
Supplement: Supplementary file 1 [file molecules-29-01822-s001.zip › Table S4.pdf]

Table S4 HPLC-ESI-MS analysis of anthocyanin extracts of *RdCHS1* over-expressing transgenic tobacco flowers

Peak number	Identifacation/tentative identification	Retention time (min)	ESI-MS (m/z)
1	Cyanidin 3-*O*-rutinoside	34.51	287.1<br>595.2
2	Cyanidin 3-*O*-(6-*O*-malonyl-beta-D-glucoside)	40.23	287.1<br>535.1
