# Supplementary figures and images for: Chalcone-Synthase-Encoding RdCHS1 Is Involved in Flavonoid Biosynthesis in Rhododendron delavayi
Source: Molecules. 2024 Apr 17;29(8):1822. doi: 10.3390/molecules29081822 (PMC11054853; doi:10.3390/molecules29081822)

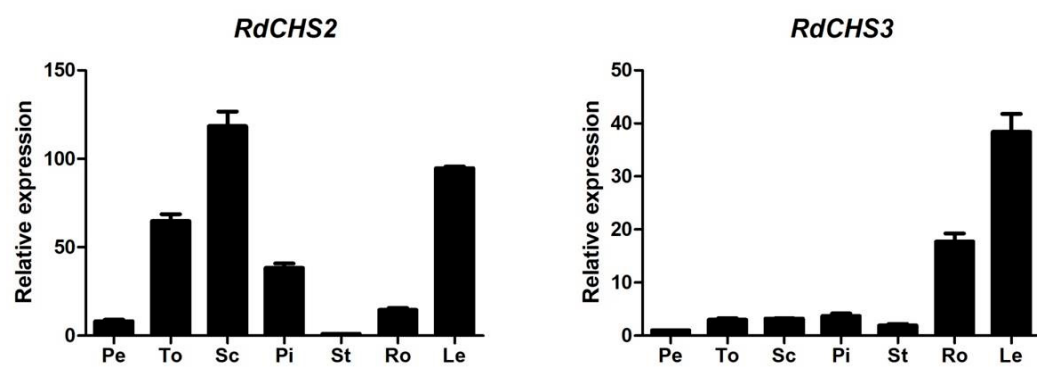

Figure S1 Expression analysis of *RdCHS2* and *RdCHS3*

Supplement: Supplementary file 1 [file molecules-29-01822-s001.zip › Figure S1.pdf]
